# Short-Term Outcomes of a Social and Emotional Learning Program: Gender-Specific Patterns in Early Adolescents

**DOI:** 10.3390/children13060805

**Published:** 2026-06-11

**Authors:** Laura Ferro, Eleonora Centonze, Mariagrazia Monaci, Giuseppe Di Maria, Stefania Cristofanelli

**Affiliations:** Department of Human and Social Sciences, University of Aosta Valley, 11100 Aosta, Italy; e.centonze1@univda.it (E.C.); m.monaci@univda.it (M.M.); g.dimaria@univda.it (G.D.M.); s.cristofanelli@univda.it (S.C.)

**Keywords:** adolescence, life skills, social and emotional learning (SEL), psychological wellbeing, response shift, gender differences

## Abstract

**Background/Objectives**: Adolescence is a crucial stage of development, in which life skills are essential for promoting psychological well-being. Social and Emotional Learning (SEL) interventions aim to develop social–emotional and relational skills that foster resilience and adaptation. Short-term effects may be nonlinear and influenced by gender differences, with possible fluctuations in self-assessments due to increased social–emotional awareness (response shift). **Methods**: This action research study involved 179 preteens (ages 11–17) from educational settings in Aosta Valley. The SEL program consisted of three experiential sessions on key life skills, led by psychologists and psychiatrists and including group activities and role-playing. Quality of life was assessed before and after the intervention using the Q-LES-Q, which measures satisfaction and well-being in the areas of social relationships, physical health, academic performance, leisure activities, and subjective feelings. Subscale scores were calculated as the mean of the corresponding items. **Results**: The results revealed nonlinear patterns: a decline in satisfaction with social relationships, which may reflect a response shift. Males reported higher initial levels and greater perceived positive effects, while females reported lower post-intervention scores, likely due to greater self-reflection and self-criticism. **Conclusions**: The data highlight the complexity of the short-term effects of SEL interventions and the importance of considering developmental trajectories and gender differences when evaluating their effectiveness.

## 1. Introduction

Adolescence is a crucial developmental stage characterized by profound cognitive, emotional, and social changes that require increasingly sophisticated skills to cope effectively with environmental demands. In this context, promoting psychological well-being is not merely about preventing distress, but also about actively developing individual and relational resources that enable adolescents to adapt flexibly and effectively to the challenges of growing up [1,2].

Among these resources, life skills are a central concept. The World Health Organization [3] defines life skills as abilities that promote adaptive behaviors, enabling individuals to overcome the challenges life presents. The WHO identifies ten life skills, grouped into three domains of competence: emotional (self-awareness, coping with emotions, and coping with stress), cognitive (decision making, problem solving, creative thinking, and critical thinking), and relational (empathy, effective communication, and interpersonal relationship skills). More recently, digital awareness has been proposed as an additional life skill [4]. In fact, if life skills are the personal abilities that enable individuals to make choices that protect their health, as suggested by the Ottawa Charter [5], digital awareness is nowadays essential to achieving this goal. The development of these skills has been linked to positive outcomes in terms of psychological well-being, social functioning, and quality of life, emerging as an important protective factor in adolescent development. Recent clinical and developmental literature has increasingly emphasized the relevance of Social and Emotional Learning (SEL) interventions not only for educational outcomes, but also for the prevention of emotional distress and the promotion of mental health during adolescence. Adolescence is recognized as a sensitive developmental period characterized by heightened vulnerability to anxiety symptoms, depressive manifestations, emotional dysregulation, interpersonal difficulties, and psychosomatic complaints, particularly following the psychosocial consequences of the COVID-19 pandemic [6,7,8]. In this context, school- and community-based SEL interventions are increasingly considered preventive public health strategies capable of strengthening protective psychosocial factors before the onset of clinically significant psychopathological conditions [9,10,11].

In recent years, numerous studies have examined the effectiveness of life skills and social–emotional learning (SEL) interventions in school settings. Meta-analyses and longitudinal studies have shown that such programs can foster social–emotional skills, prosocial behaviors, and psychological well-being, with effects that can endure over time [12,13]. Recent meta-analyses and umbrella reviews have shown that SEL programs can positively influence emotional regulation, self-efficacy, resilience, peer relationships, stress management, and psychological well-being across different developmental stages [14,15]. Importantly, the effects of SEL interventions appear to extend beyond academic functioning, contributing to reductions in internalizing symptoms, emotional distress, and maladaptive coping strategies [16]. Emerging evidence also suggests that interventions focused on emotional awareness and interpersonal competence may improve adolescents’ ability to recognize emotional states, regulate stress responses, and seek social support more effectively, all of which are clinically relevant protective factors for mental health trajectories [15]. However, these effects are not always linear or uniform across different domains of functioning.

From a neurodevelopmental perspective, adolescence represents a phase of substantial maturation of socio-cognitive and emotional processing systems. Contemporary developmental neuroscience suggests that the imbalance between heightened emotional reactivity and still-maturing executive and regulatory systems may increase adolescents’ sensitivity to stress and social evaluation [14]. SEL interventions may, therefore, support adaptive developmental processes by strengthening emotional regulation, reflective functioning, and interpersonal competence during this particularly sensitive developmental window [17].

In particular, the most recent research suggests that life skills interventions can produce complex and sometimes counterintuitive outcomes in the short term. In fact, increased emotional and social awareness may lead to a recalibration of self-assessment criteria, resulting in a temporary decrease in self-report scores in certain areas, particularly those related to interpersonal relationships [9]. This phenomenon, known as a response shift, reflects a change in internal evaluation criteria rather than an actual decline in skills [18].

Furthermore, recent clinical literature suggests that short-term changes following psychosocial interventions should be interpreted cautiously, especially when self-report measures are used. Increased self-awareness and emotional literacy may initially produce more critical self-evaluations, particularly in relational domains, resulting in temporary reductions in perceived well-being despite ongoing developmental gains [19]. This perspective is coherent with response shift models and with developmental theories emphasizing the progressive reorganization of self-concept and interpersonal understanding during adolescence [20,21].

These mechanisms are particularly significant during preadolescence, a period in which the development of social cognition and the self-system leads to an increasing capacity for reflection and critical analysis of interpersonal relationships and one’s own skills [22,23,24]. From this perspective, any negative changes in self-reported assessments can be interpreted as indicators of an ongoing developmental process characterized by greater complexity and differentiation in self-representation.

Another clinically relevant aspect concerns gender differences in adolescent psychological well-being. Epidemiological studies consistently show that adolescent girls report higher levels of internalizing symptoms, emotional distress, psychosomatic complaints, perceived loneliness, and stress-related difficulties compared to boys [25,26]. These differences become particularly pronounced during early adolescence, likely due to interactions among biological maturation, socialization processes, self-evaluative tendencies, and relational sensitivity [27]. Consequently, several authors have suggested that gender may influence not only baseline levels of psychosocial functioning, but also the way adolescents perceive and report changes following SEL interventions [28].

Furthermore, evidence suggests that individual skills generally respond more readily to short-term interventions, whereas interpersonal skills—which are rooted in group dynamics and complex contexts—require more time to develop [25]. This can lead to a temporary discrepancy between individual improvements and relational perceptions, highlighting the dynamic and nonlinear nature of social–emotional development.

In line with these theoretical perspectives, recent studies on life skills programs show varying outcomes across domains and groups, highlighting how some skills (such as self-efficacy and prosocial behaviors) improve more rapidly, while other dimensions, such as self-perceptions and relational evaluations, may fluctuate in the short term [14,15,21]. These findings support the idea that the observed changes should be interpreted within the context of complex and progressive developmental trajectories.

Taken together, these findings support the importance of examining SEL outcomes within a multidimensional and developmental framework that considers not only symptom reduction or immediate improvements, but also evolving processes of self-awareness, emotional differentiation, and relational reorganization. Within this framework, the present action research study fits within this context and aims to evaluate the effectiveness of a life skills-based intervention, grounded in the WHO framework [29], in promoting psychological well-being and psychosocial skills among a sample of preteens. In accordance with the guidelines set forth in the Glossary of Health Promotion [30], the actual SEL program was based on an experiential approach centered on practical activities, which stimulated group discussions and peer-to-peer exchanges, fostering shared reflection among preteens and with the adult group facilitators. Participatory and experiential approaches have been shown to be particularly effective in fostering life skills during adolescence [31].

### 1.1. The Present Study

Building on the available evidence, this action research study aimed to improve satisfaction across various areas of life—including physical health, emotional well-being, peer relationships, school activities, and leisure time—among a group of adolescents. The study examined short-term changes following a social–educational learning program consisting of three sessions, during which the students participated in activities designed to encourage reflection on the WHO’s 10 life skills and digital literacy.

#### Context and Aims

The project was launched as part of the agreement between the University of Valle d’Aosta and the Autonomous Region of Valle d’Aosta, Department of Health, Healthcare, and Social Policies, which funded the research action program.

This partnership stems from the Region’s commitment to addressing the needs of young people in the Aosta Valley in order to prevent psychological distress and promote well-being and mental health, particularly in light of recent surveys involving young people in the Region. Among these is the HBSC (Health Behavior in School-Aged Children) study, conducted nationwide every four years; it is an international multicenter study carried out in collaboration with the WHO. In the Aosta Valley, the 2022 HBSC study found that 44% of young people aged 11 to 17 experienced physical or psychological symptoms every day, with a higher prevalence among females than males. Furthermore, 38.3% of adolescents reported a low level of overall psychological well-being. Socially speaking, 12.1% of adolescents reported feeling lonely most of the time, and 2.5% reported feeling lonely all the time. Schoolwork also appears to be a source of stress: 62.1% of young people reported feeling very or somewhat stressed by their schoolwork. In all the areas mentioned, girls exhibited higher levels of distress, with a greater incidence of psychosomatic symptoms, perceived loneliness, and school-related stress [32]. A further survey conducted among students in the Aosta Valley between May and July 2022 [33] revealed that, from the perspective of young people, there are six main issues to be addressed: the first, which stands out above the rest, is a lack of peace of mind and self-confidence (20.1%); followed by excessive use of devices (11.2%), difficulty relating to peers (10%), academic failure (9.9%), body image and sexuality (9.6%), and difficulty relating to adults (9%). Finally, the young people surveyed viewed their future as uncertain and a source of anxiety: about a quarter of them looked to the future with pessimism, and 11% had a cynical outlook.

## 2. Materials and Methods

### 2.1. Study Design

**Figure 1:**
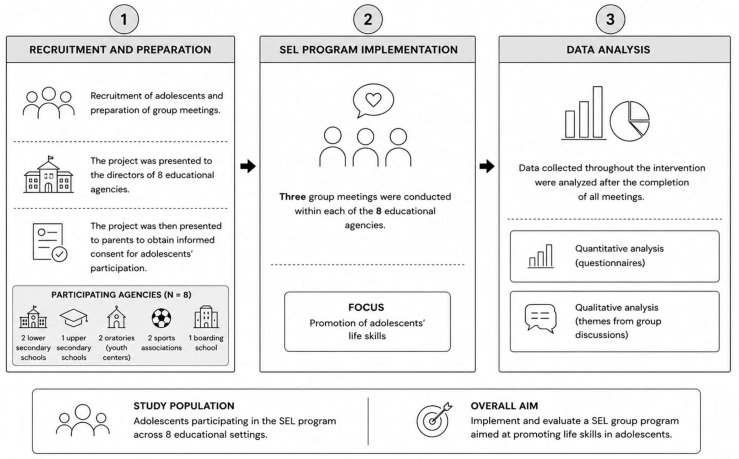
Study design.

The present study was designed as a research–intervention project conducted in real-world educational and community settings. The study focused on the implementation of a Social and Emotional Learning (SEL) program aimed at promoting adolescents’ life skills through group-based activities. Figure 1 shows the study design.

The research was structured into three main phases. The first phase involved participant recruitment and preparation of the intervention activities. Eight educational agencies participated in the project (two middle schools, one high school, one boarding school, two sports clubs, and two religious groups) in the Aosta Valley, a region of northwestern Italy. The design research was initially presented to the legal representatives of the institutions involved; following approval by the organization, the study was presented to the preteens’ parents. Participation was not mandatory; the families who chose to join the study provided their written consent. See Section 2.2 for a description of participants.

The second phase involved the implementation of the SEL program. Three group meetings were conducted within each educational agency, focusing on the promotion of adolescents’ life skills (see Section 2.3).

The third phase involved the analysis of the collected data following the completion of the intervention activities. See Section 2.5 for a deeper description of the measures collected and Section 3 for a description of the results.

### 2.2. Participants

A total of 179 respondents (60 females, 5 preferred not to answer; ages ranging from 11 to 17 years) constituted the sample at T1, before the SEL program.

At T2, after the intervention, 60 respondents (20 females, 1 preferred not to answer; ages ranging from 11 to 16 years) were matched to the responses at T1 through an anonymous code consisting of the last 4 digits of their parents’ phone number. Unfortunately, attrition between T1 and T2 was substantial. Several practical factors inherent to the applied nature of the intervention contributed to participant attrition over time. In school settings, questionnaires were generally completed during scheduled sessions with access to institutional devices, although absenteeism and some difficulties in matching anonymous participant codes across waves led to additional data loss. In contrast, participants in more informal community settings completed the follow-up questionnaire remotely after the intervention, resulting in markedly lower response rates.

A participant feedback questionnaire on the intervention was administered at the end of the third and final meeting, and 72 (23 females, 3 preferred not to answer) participants completed it. The questionnaire was given to all the participants who attended the last meeting, even those who had not attended the first. For this reason, the number of respondents was higher than the number of those who completed the Q-LES-Q [34] before and after the meetings.

Although this was an action research study rather than a research study, and we could not control the number of participants, we conducted a sensitivity analysis. A post hoc power analysis of the difference between the two independent means (the two groups of males and females before the SEL program) was conducted using G*Power 3.1.9.7. With this sample size at T1 (N = 179) and 80% power (α = 0.05), the critical t-value was found to be 1.65. Table 1 shows the t-values for the conducted analyses, which, in cases of significant differences, exceeded the critical t-value. For our second analysis, after the SEL program, a post hoc power analysis for MANOVA with repeated measures and a sample size at T2 of N = 60, with an effect size of 0.25 of (α = 0.05) and two measurements and two groups, indicated a critical F of 4.00; again, the critical F-value was reached when the results were significant; the observed power is also reported.

Data protection officers verified whether data regulation rules were complied with. The study was conducted in accordance with the Declaration of Helsinki and approved by the Ethics Committee of the University of Aosta Valley. The survey was designed to be easily completed in 8–10 min.

### 2.3. The Socio-Emotional Learning (SEL) Program

For each of the 8 educational agencies, three sessions were held, each lasting about two hours (for a total of six h), focusing on life skills. All the sessions were led by a psychologist and a psychiatrist. For each educational agency, the group consisted of an average of 15 teenagers (min = 8; max = 22).

The main interactive tool used throughout the program was a backpack containing 10 items representing the following life skills: decision making, problem solving, creative thinking, critical thinking, empathy, effective communication and interpersonal relationship skills, coping with emotions, coping with stress, self-awareness, and digital awareness.

At the first meeting, the facilitators introduced the backpack to the participating students and explained what life skills are. One by one, the children took turns picking out an object, and together they tried to match it with a life skill. It should be noted that the students had just learned the general definition of “life skills,” but they were not familiar with the list of life skills. By the end of the first session, the students had selected the top five items and discussed the corresponding life skills.

The second session was conducted in the same way, allowing the students to explore the other five life skills. A new activity was then introduced to help the students deepen their self-awareness: they were asked to reflect on their desires (short-term and/or medium- to long-term) and to consider possible strategies for fulfilling them.

During the third and final session, two additional activities were introduced: the scales game (based on critical thinking) and the puzzle of manipulative versus constructive criticism (based on effective communication and interpersonal relationship skills). In the first activity, the facilitators drew a two-pan scale on a whiteboard: one pan was labeled with a need (e.g., autonomy), while the other remained empty; the students’ task was to identify the need that contrasted with the one highlighted by the adult (e.g., protection). The second activity, conducted after defining manipulative and constructive criticism [35,36], involved a role-playing experience in which participants had to distinguish between manipulative and constructive criticism.

### 2.4. Aims and Hypotheses

The aim of this study is to contribute to our understanding of the processes through which life skills act as protective factors for psychological well-being in adolescence, with a particular focus on short-term changes and individual differences in intervention outcomes. In particular, it is expected that:

• The SEL program, based on life skills, improves perceived satisfaction, particularly in the areas of social relationships and subjective feelings, which stand to benefit most from the intervention, since life skills enhance interpersonal and communication skills as well as emotional regulation. However, it is possible that these improvements may not become apparent in the short term due to the so-called response shift effect [18]. In fact, it is possible that satisfaction levels may decline in the short term, not so much because of an actual decline in quality of life, but rather due to a more self-critical—yet also more realistic—assessment stemming from greater awareness [12,24].
• There will be gender differences in the effects of the SEL program, in particular, because girls tend to be more reflective and self-critical, while boys tend to be more overconfident in their self-assessments [19,25], we expect higher satisfaction levels among boys and lower levels among girls.

### 2.5. Measures

The Quality of Life Enjoyment and Satisfaction Questionnaire (Q-LES-Q) [34,35] is a self-report questionnaire that measures the degree of pleasure and satisfaction experienced over the past week across various areas of daily life, each represented by a specific subscale. The original version of the instrument consists of 93 items, 91 of which can be grouped into 8 scales: Physical Health (13 items), Subjective Feelings (14 item), Leisure Time Activities (6 items), Social Relationships (11 items), General Activities (14 items); there are also three scales to be completed only if they apply to the respondent’s life situation: Work (13 items), Household Responsibilities (10 items), and Academic Performance (10 items). In addition to these items, two others are evaluated separately: one measures Satisfaction With The Treatment, and the other measures Overall Satisfaction. Items are rated on a Likert-type scale ranging from 1 (=not at all, never) to 5 (=always or almost always); the higher the score, the higher the level of satisfaction. The internal consistency (Cronbach’s alpha) of the scales proposed by the Q-LES-Q ranges from 0.90 to 0.96, and the test–retest reliability (interclass correlation coefficient) ranges from 0.63 to 0.89 [34,35]. The scale demonstrated good internal consistency (Cronbach’s alpha > 0.80 for each subscale) even in the Italian context [34]. In this study, items related to the General Activities scale were excluded because they included questions about sexual activity, which were not appropriate for the intervention’s target participants; similarly, only the Academic Performance scale was included, while the scales related to work and household duties were not. The single item regarding satisfaction with the treatment was also not included because it is not applicable to the present research context. In this study as well, the scales demonstrated good internal consistency (Cronbach’s alpha > 0.80 for each scale), as shown in Table 1, confirming the findings of the Italian validation study [34].

In summary, this study utilized the following five subscales from the Italian adaptation of the Q-LES-Q [34]:

• Physical Health, 13 items, (e.g., “Over the past week, how often… did you feel full of energy?”);
• Subjective Feelings, 14 items (e.g., “Over the past week, how often… did you feel satisfied with your life?”);
• Academic Performance, 10 items (e.g., “Over the past week, how much time… did you spend on your schoolwork?”);
• Leisure Activities, 6 items (e.g., “Last week, when you had some free time, how much of it did you spend on hobbies?”);
• Social Relationships, 11 items (e.g., “Over the past week, how often…did you enjoy talking with or spending time with friends or family?”)

The five factors were constructed by summing the scores obtained on all the items comprising each scale [34,35].

## 3. Results

Table 1 presents descriptive data and the intercorrelations among the five subscales of the Q-LES-Q, measured at Time 1, before the SEL program activities. The highest level of satisfaction was for Social Relationships, followed by Physical Health, while the lowest level was for Academic Performance. The correlations among the subscales are consistently high-to-moderate and significant, with the strongest correlations found in the relationship between Physical Health and Subjective Feelings.

A series of *t*-tests was used to compare male and female responses on the subscale scores (the five respondents who preferred not to specify their gender have been excluded from the analysis).

The results of the comparisons are presented in Table 2.

For Academic Performance and Social Relationships, the gender differences did not reach significance, whereas for Physical Health, Subjective Feelings, and Leisure Activities, the differences were significant; in all three domains, males reported being more satisfied.

### 3.1. After the SEL Program

To examine changes following the intervention and potential gender differences, five separate repeated-measures mixed ANOVAs were conducted for the Q-LES-Q subscales. Time (pre-intervention vs. post-intervention) was entered as the within-subject factor and gender as the between-subject factor. This analytic approach allowed us to evaluate overall changes across time, gender differences, and Time × Gender interaction effects for each domain of quality of life.

Prior to the analyses, assumptions were examined. Because the within-subject factor included only two measurement occasions, the assumption of sphericity was automatically met. Descriptive statistics and effect sizes (partial η^2^) are reported for all significant effects.

**Figure 2:**
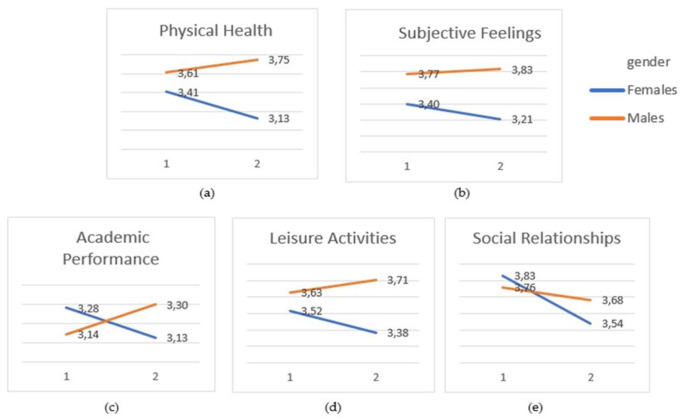
Females’ and males’ patterns over time of the means for (**a**) Physical Health; (**b**) Subjective Feelings; (**c**) Academic Performance; (**d**) Leisure Activities; (**e**) Social Relationships.

Figure 2 shows the mean patterns. The main within-subject factor with repeated measures collected before and after the intervention emerged as significant only for the Social Relationships subscale (F_(57,1)_ = 4.51, *p* = 0.039, η^2^_p_ = 0.072, observed power 0.43), with a medium effect size as indicated by the partial eta square, showing satisfaction within the domain of social relationships decreased across all the respondents. Gender emerged as significant for the Physical Health (F_(57,1)_ = 5.7, η^2^_p_ = 0.091, observed power 0.65), and for Subjective Feelings (F_(57,1)_ = 7.4, *p* = 0.009, η^2^_p_ = 0.12, observed power 0.76), subscales, in both cases with higher scores for males and a large effect size. For Physical Health, the main effect of gender was also qualified by a significant interaction (F_(57,1)_ = 6.5, *p* < 0.01, η^2^_p_ = 0.10, observed power 0.71), indicating that the SEL program had a positive impact on males but a negative impact on females (see Figure 2). The interaction was only marginally significant for Academic Performance (F_(57,1)_ = 3.3, *p* = 0.075, η^2^_p_ = 0.055, observed power 0.43), albeit with a similar pattern, with an improvement in males and a worsening in females, which was also observed for Subjective Feelings and Leisure Activities, although it did not approach statistical significance (probably because of the small number of females). The *t*-tests conducted as post hoc analyses confirmed significant gender differences in Physical Health and Subjective Feelings at T1 and T2, with males consistently reporting higher values. Follow-up independent-samples tests confirmed the gender differences observed in the mixed ANOVAs. Specifically, males reported significantly higher Subjective Feelings scores at both T1 (t(57) = −2.01, *p* = 0.049) and T2 (t(57) = −2.84, *p* = 0.006), as well as higher Physical Health scores at T2 (t(57) = −3.10, *p* = 0.003).

### 3.2. Satisfaction with the Socio-Educational Intervention

Following the intervention, the level of appreciation and satisfaction with the socio-educational program was assessed using eight questions. The relative means and gender comparisons are reported in Table 3. We observed that higher satisfaction was associated with the clarity of information communication, followed by satisfaction with the activities carried out and the acquisition of useful life-planning skills.

In the gender comparisons, males were once again found to express significantly greater satisfaction.

They believed, to a greater extent than female participants, that the activities of the intervention/program had changed their attitude towards mental health, that they had acquired useful life skills, and that they had improved their relationships with others.

## 4. Discussion

This action research study adds to the growing body of literature on social and emotional learning (SEL) by examining its short-term effects during preadolescence and highlighting the complex, nonlinear, and context-dependent nature of the observed outcomes.

The main findings indicate a decline in satisfaction with social relationships among all participants following participation in the SEL program. However, only male participants showed improvements in Physical Health and Leisure Activities, domains in which they had already reported higher satisfaction than females before the intervention. Male participants also reported greater overall satisfaction with the SEL program and were more likely to perceive improvements in life skills and interpersonal relationships.

In line with the World Health Organization’s framework on life skills and contemporary models of social–cognitive development, the findings suggest that social–emotional learning (SEL) interventions may not produce immediate or uniform improvements. Instead, they may trigger dynamic processes of cognitive, emotional, and relational reorganization.

At the same time, a reduction in perceived satisfaction with social relationships emerged across the sample. Rather than indicating a detrimental effect of the intervention, this pattern may reflect changes in subjective evaluation processes.

Specifically, this decrease may be interpreted as being consistent with the response shift phenomenon [18], whereby increased socio–emotional awareness leads to a recalibration of internal standards and evaluative criteria. As adolescents become more capable of recognizing complexity, ambivalence, and relational nuances, they may become more critical and discerning in their self-evaluations. However, because response shift was not directly assessed in the present study, this interpretation should be considered as a plausible explanatory framework rather than an empirically demonstrated mechanism.

This interpretation is further supported by developmental models of social cognition [36] and self-system development [37,38], which conceptualize preadolescence as a critical transitional phase marked by rapid advances in metacognitive capacity, reflective thinking, and interpersonal sensitivity.

An additional explanatory dimension concerns the differential temporal dynamics of skill acquisition.

Intrapersonal competencies, such as self-awareness and emotional regulation, may respond more readily to brief interventions, whereas interpersonal competencies generally require longer periods to consolidate [24]. This discrepancy may cause a temporary misalignment between improvements at an individual level and the perceived quality of relationships.

The gender differences identified further contribute to the complexity of the findings. Male participants reported higher baseline levels of perceived well-being and greater perceived benefits from the intervention, including improvements in life skills and relationships. These findings are consistent with previous literature on gender differences in emotional socialization and self-evaluative styles [17,19,25]. Female participants, by contrast, displayed more varied and partially declining trajectories in some domains, particularly Physical Health and Academic Performance.

This pattern may reflect heightened metacognitive sensitivity and stronger engagement in evaluative recalibration processes, which are consistent with, although not sufficient to demonstrate, the mechanisms of response shifts documented in previous SEL research [18,24].

These findings should not be interpreted as evidence of differential intervention effectiveness.

Taken together, the findings support the interpretation of SEL outcomes within a framework of nonlinear developmental trajectories, in which behavioral improvements, perceptual changes, and shifts in evaluative standards may unfold asynchronously.

This perspective is consistent with Self-Determination Theory [2], which conceptualizes psychological development as a process of progressive internalization and reorganization of experience, often characterized by temporary instability prior to consolidation into more autonomous and integrated forms of functioning.

### Limitations and Future Research

From a methodological standpoint, the study has several limitations, including the exclusive use of self-report measures, the absence of multi-informant and qualitative data, and the failure to incorporate an explicitly gender-responsive design. Furthermore, the lack of a longer-term follow-up makes it impossible to assess the duration or changes in the program’s effects over the medium term. Another limitation concerns the absence of a control group, which limits causal interpretation of the observed effects; therefore, the changes observed cannot be unequivocally attributed to the intervention itself. However, this methodological constraint should be interpreted in light of the applied and ecological nature of the research–intervention design, which posed substantial challenges for the recruitment and management of comparable non-intervention groups. Another important limitation concerns the high attrition rate between T1 and T2. Participant loss was primarily due to practical and organizational constraints inherent to the implementation of the intervention across diverse real-world contexts. In particular, follow-up participation was higher in structured settings (e.g., schools) and lower in informal community contexts, where participants completed the follow-up assessment remotely after the intervention. Due to the anonymized data collection procedure and incomplete linkage of participant codes across waves, it was not possible to systematically compare baseline characteristics of participants who completed both assessments and those lost to follow-up. Therefore, the presence of attrition bias cannot be ruled out. The final longitudinal sample may therefore differ in its level of engagement and compliance with study procedures, which should be considered when interpreting the findings.

Taken together, these factors suggest that caution should be exercised when interpreting the results. Future action research studies should therefore incorporate follow-up assessments and complementary data sources, including behavioral observations and qualitative methods, to disentangle temporary evaluative recalibration from enduring changes in socio-emotional competencies.

An additional limitation concerns the relatively small subsample of participants who completed the Q-LES-Q both before and after the intervention, particularly the lower number of female participants. Consequently, some analyses were characterized by limited statistical power, and the observed gender differences should be interpreted with caution. Future studies should seek to replicate these findings in larger and more balanced samples to clarify whether gender-specific patterns of response to SEL interventions are robust and generalizable.

In addition, the design of gender-sensitive interventions appears crucial for maximizing the effectiveness of SEL programs and preventing unintended disparities in outcomes.

## 5. Conclusions

The present study highlights the importance of adopting longitudinal, multi-method, and multi-informant approaches in the evaluation of SEL interventions, in order to distinguish between temporary declines linked to increased awareness and more stable changes in skills [6,7]. Exclusive reliance on short-term pre–post self-report measures may lead to an overestimation of transient negative effects or fail to capture delayed and more stable benefits. In addition, SEL programs should be tailored more specifically to participants, particularly with regard to gender.

In conclusion, our findings suggest that SEL programs should not be viewed as linear interventions that produce immediate improvements. Instead, they should be considered as complex developmental processes that promote awareness, restructuring, and the gradual integration of socio-emotional skills over time.

## Figures and Tables

**Table 1:** Descriptive data and intercorrelations among the five Q-LES-Q subscales measured at Time 1.

	Mean	SD	Cronbach Alpha		2	3	4	5
1. PH	3.53	0.72	0.87		0.80	0.61	0.46	0.57
2. SF	3.61	0.77	0.92			0.59	0.51	0.60
3. AC	3.15	0.82	0.90				0.36	0.43
4. LA	3.55	0.68	0.73					0.45
5. SR	3.73	0.62	0.82					—

*Note*. PH: Physical Health, SF: Subjective Feelings, AC: Academic Performance, LA: Leisure Activities, SR: Social Relationships. All correlations are significant at *p* < 0.001.

**Table 2:** *T*-test comparisons of gender on the Q-LES-Q subscale at T1, before the intervention.

	Females (N = 60)		Males (N = 114)		t	*p*
	Mean	SD	Mean	SD		
PH	3.36	0.75	3.66	0.66	−2.687	**0.004**
SF	3.36	0.76	3.78	0.70	−3.679	**0.001**
AP	3.14	0.80	3.18	0.81	−0.335	0.369
LA	3.43	0.74	3.63	0.65	−1.785	**0.038**
SR	3.69	0.63	3.77	0.61	−0.842	0.200

*Note*. PH: Physical Health, SF: Subjective Feelings, AP: Academic Performance, LA: Leisure Activities, SR: Social Relationships. In **bold** significant differences at *p* < 0.05.

**Table 3:** Global satisfaction and *t*-test comparisons for gender on the level of satisfaction with the intervention.

	Total		Males (N = 46)		Females (N = 23)		*p*
	Mean	SD	Mean	SD	Mean	SD	
Was information communicated clearly during the project activities?	3.65	1.01	3.67	0.99	3.74	1.05	0.401
Did the activities you participated in encourage reflection and discussion with your classmates?	2.99	1.11	3.04	1.11	2.91	1.16	0.327
Has your attitude toward mental health changed as a result of the activities you participated in?	2.50	1.21	2.70	1.24	2.17	1.11	0.047
To what extent do you feel you have acquired skills that are useful for your life planning?	3.10	1.04	3.33	1.03	2.65	0.93	0.005
Did participating in the project help you improve your relationships with others?	2.58	1.21	2.76	1.25	2.22	0.95	0.036
How satisfied are you with the activities you carried out during the project?	3.26	1.24	3.35	1.23	3.09	1.20	0.203
How much would you like to do this project again?	3.06	1.28	3.13	1.22	3.00	1.38	0.345
How likely are you to recommend this project to people you know?	3.32	1.05	3.43	1.09	3.17	0.98	0.168

## Data Availability

The dataset is available on request from the authors.
